# Recent advances in early diagnosis and treatment of T1D with miRNAs

**DOI:** 10.3389/fendo.2025.1582963

**Published:** 2025-05-15

**Authors:** Xinling Zhang, Yuting Qiu, Dong-ang Liu, Ruiyao Hu, Shiyu Chen, Yue Xu, Keyi Chen, Jinghua Yuan, Xiaoping Li

**Affiliations:** Key Laboratory of Artificial Organs and Computational Medicine in Zhejiang Province, Shulan International Medical College, Zhejiang Shuren University, Hangzhou, China

**Keywords:** T1D, miRNAs, biomarkers, diagnosis, complications, treatment

## Abstract

Type 1 diabetes (T1D) is an autoimmune disease characterized by T cell-mediated destruction of pancreatic β-cells and is one of the most common chronic diseases in adults and children. In recent years, the incidence of T1D has been increasing worldwide. Currently, the diagnosis of T1D relies on clinical manifestations and autoantibody detection, with a lack of early predictive biomarkers. MicroRNAs (miRNAs), as crucial post-transcriptional regulatory factors, which are involved in various biological processes, including cell division, proliferation, differentiation, development, and metabolism. Additionally, miRNAs participate in the regulation of inflammatory complications in T1D, and their aberrant expression is closely associated with the disease. The stability of miRNAs makes them potential candidates for early diagnostic biomarkers and therapeutic targets in T1D. This paper discusses the pathogenesis of T1D and the potential applications of miRNAs in early diagnosis and interventional therapy. It provides references for advancing precision diagnosis and personalized treatment strategies through more profound miRNA research in the future.

## 1 Introduction

With the improvement in living standards and changes in lifestyle, diabetes has become a health issue that cannot be ignored. Diabetes is classified into type 1 diabetes (T1D) and type 2 diabetes (T2D) based on etiology. T1D is a chronic autoimmune disease characterized by T cell-mediated, specific destruction of pancreatic β-cells, leading to absolute insulin deficiency and hyperglycemia (1, 2). T1D not only affects adults but is also one of the most prevalent chronic childhood diseases. An extensive array of data indicates that the incidence of T1D has been increasing significantly worldwide in recent years (3). New modeling data indicate that by 2040, the number of individuals with T1D is projected to reach between 13.5 and 17.4 million. Notably, the most significant relative growth is expected to occur in low-income and lower-middle-income countries (4). Deaths caused by T1D are primarily due to delayed diagnosis and treatment (4). Currently, the diagnosis of T1D still relies mainly on clinical manifestations, and there is a lack of sensitive and effective biomarkers for early prediction of T1D. Both cellular and humoral immune responses are dysregulated in T1D, and several genetic loci are associated with the risk of T1D. This implies a complex multifactorial pattern of inheritance for T1D (5). MicroRNAs (miRNAs), as important post-transcriptional regulatory factors, dynamically regulate the growth and development of T and B lymphocytes. Abnormal increases or decreases in miRNAs can affect the onset and progression of T1D. Therefore, miRNAs play a key regulatory role in the pathogenesis of T1D. As a result, certain miRNAs are expected to serve as novel biomarkers for early diagnosis of T1D and as new therapeutic targets. We discuss a number of established and emerging predictive and prognostic biomarkers, including markers of pancreatic islet function that can improve strategies to measure the outcome of therapeutic interventions. The article focuses on the latest research progress of inflammation-related biological markers in T1D, especially the role of miRNAs in the early diagnosis and interventional treatment of T1D (6). It provides essential references and valuable insights for advancing precision diagnosis and personalized treatment strategies. Future research focusing on miRNAs will be particularly important for biomarker discovery, therapeutic target identification, and molecular pathway regulation.

## 2 Overview and Pathogenesis of T1D

T1D is an organ-specific autoimmune disease characterized by the infiltration and attack of pancreatic β-cells by T cells and other immune cells, leading to β-cell destruction and subsequent insulin deficiency (7). T1D is one of the most common chronic diseases in children and adolescents. In recent years, the incidence and prevalence of T1D have gradually increased, with significant geographical disparities in incidence (8, 9). Insulin is a key hormone that regulates metabolism and growth, promoting glucose uptake, glycogen synthesis, fatty acid synthesis, and amino acid uptake while inhibiting lipolysis and maintaining electrolyte balance. Therefore, individuals with T1D require lifelong insulin replacement therapy (10, 11).

**Figure 1:**
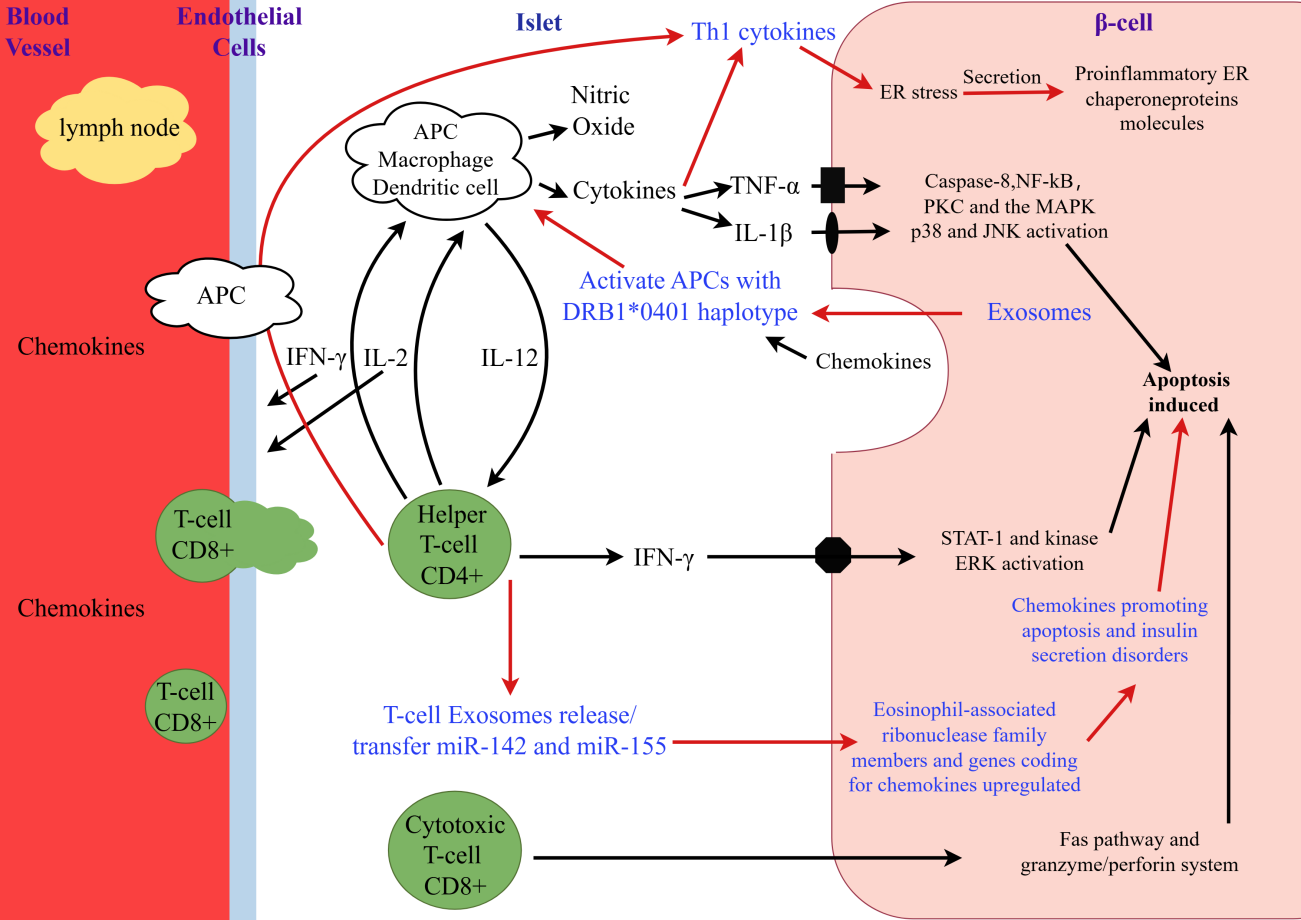
The role of exosomes in the pathogenesis of type 1 diabetes. This figure was drawn by Figdraw. During the early phase of insulitis, local antigen-presenting cells (APCs) are activated and recruit CD4+ helper T cells from the pancreatic lymph nodes, thereby releasing chemokines/cytokines. CD4+ helper T cells induce APCs to secrete cytokines and nitric oxide and stimulate endothelial cells to release chemokines, thereby activating CD8+ cytotoxic T cells. β-cells that respond to viral infections or secrete chemokines in response to cytokines further stimulate and activate immune cells, leading to the activation of the Fas pathway and the granzyme/perforin system, which induces β-cell apoptosis. Additionally, IL-1β, TNF-α, and IFN-γ directly bind to their respective receptors on the surface of β-cells and induce apoptosis. The number following the asterisk (*) (0401) indicates the specific allele or variant of the gene.

Initially, the etiology of T1D was considered to be a purely autoimmune disease caused by T cell-mediated destruction of pancreatic β-cells. However, with ongoing research into the etiology and pathogenesis of T1D, it is now understood that the disease results from the interplay of multiple factors, including genetic susceptibility, the immune system, environmental factors, gut microbiota, and metabolic processes (12). **Figure 1** illustrates the role of exosomes in the pathogenesis of T1D. Firstly, genetic defects are recognized as the foundation for the development of T1D. The disease is associated with genetic susceptibility, particularly with specific human leukocyte antigen (HLA) alleles (13). Secondly, T1D may arise from defects in the immune system. Individuals with T1D often have multiple autoantibodies in their bloodstream. The presence of circulating pancreatic autoantibodies indicates an increased risk of developing T1D or the presence of the disease. These antibodies include islet cell antibody (ICA), insulin antibody (IAA), glutamic acid decarboxylase antibody 65 (GAD65), insulinoma-associated protein-2 (IA-2), and zinc transporter 8 (ZnT8). The number and titer of detected antibodies are directly correlated with the risk of developing T1D (14). Thirdly, in high-risk populations, viral infections or other environmental factors can trigger the autoimmune destruction of β-cells (15). Viruses are important triggers for the onset of T1D, with human enteroviruses (HEV) being particularly implicated in initiating pancreatic autoimmunity and clinical disease (16). Fourthly, the gut microbiota may be an important modulator in the pathogenesis of T1D. Studies have shown that individuals with T1D have dysbiosis of the gut microbiota, characterized by reduced microbial diversity and disrupted microbial community structure (17). Recent studies have shown that, although the rapid loss of insulin secretion from β cells occurs a few months before clinical onset, evidence of β-cell dysfunction may have existed years earlier (18). β-cell autophagy is crucial for cellular survival and function. Defective β-cell autophagy can induce ER stress, alter antigen-processing pathways, and enhance MHC-I/HLA-I presentation to immune cells, rendering β cells more susceptible to immune attack and destruction (19). Furthermore, the activation of intrinsic β-cell pathways due to autophagy defects may trigger autoimmunity by forming neoantigens or may independently accelerate autoimmune-mediated β-cell death (18).

## 3 MicroRNAs

miRNAs are single-stranded RNA molecules composed of 19 to 25 nucleotides and belong to a family of endogenous non-coding RNAs (ncRNAs) that do not encode proteins but regulate protein levels through post-transcriptional mechanisms. Among all RNAs, the majority of ncRNAs cannot encode proteins but instead exert their functions by regulating gene expression at the post-transcriptional level. miRNAs negatively regulate gene expression by binding to the 3’-untranslated region (3’-UTR) of target mRNAs, leading to mRNA cleavage or translational repression. This process influences protein translation and is involved in various physiological and pathological processes in the body. Studies have shown that miRNAs regulate the expression of immune system-related genes. They dynamically control the development of T and B lymphocytes, and participate in cell differentiation, innate immune responses, and the regulation of immune cell signaling pathways. Aberrant miRNA expression can lead to autoimmune reactions and contribute to the development of T1D (20). Additionally, miRNA-mediated gene regulation is essential for normal physiological processes, including cell cycle progression, cell differentiation, and cell death. Therefore, targeting miRNAs to modulate immune responses offers a novel therapeutic approach for T1D. Their localization determines effective gene regulation by miRNAs, the level of target mRNAs, and the interaction between miRNAs and mRNAs, ultimately resulting in the formation of functional mature miRNAs that regulate protein expression. Inflammatory cytokines such as interleukin-1β (IL-1β), tumor necrosis factor-α (TNF-α), and interferon-γ (IFN-γ), as well as pancreatic autoantibodies such as ICA, IAA, and ZnT8, are classic markers of T1D but lack specificity. Therefore, there is an urgent need to identify new biomarkers for the early detection of T1D. However, miRNAs are emerging as a promising area for further exploration in T1D research and may potentially serve as future early diagnostic markers for T1D.

## 4 The role and impact of inflammatory factors and miRNAs in T1D

Inflammatory factors and miRNAs play significant roles in the pathogenesis and disease progression of T1D. Research indicates that miRNAs modulate pancreatic β-cell function and immune responses, thereby influencing the progression of T1D (6). For example, miR-30b and miR-101a are crucial in cytokine-mediated β-cell dysfunction, reducing proinsulin production and increasing β-cell death. Additionally, miR-146a is upregulated in individuals with T1D, targeting inflammation-related genes such as tumor necrosis factor receptor-associated factor 6 (TRAF6) and interleukin-1 receptor-associated kinase 1 (IRAK1), thereby amplifying inflammatory responses. These inflammatory processes not only drive the development of T1D but are also associated with various complications, including diabetic nephropathy, retinopathy, and cardiovascular and cerebrovascular diseases. These complications can severely affect patients’ quality of life and may lead to life-threatening diabetic ketoacidosis in severe cases. Therefore, in-depth investigation of the mechanisms underlying the roles of inflammatory factors and miRNAs may facilitate the development of early diagnostic biomarkers and therapeutic targets, ultimately improving the prognosis of patients with T1D (21).

## 5 miRNAs and T1D

To date, the risk assessment of T1D using autoantibodies has relied on the number of autoantibodies present. However, there is significant variation in the progression rate among individuals with multiple autoantibodies (22). This indicates that although islet autoantibodies play a crucial role in risk assessment, they may not be the most effective biomarkers for monitoring therapeutic efficacy (23). Therefore, there is an urgent need for more suitable biomarkers for pre-onset progression studies and treatment monitoring in T1D. miRNAs, as biomarkers, play a key role in the pathogenesis of T1D by regulating gene expression. These miRNAs are involved in immune regulation, cell proliferation, and insulin processing, thereby contributing to a deeper understanding of T1D mechanisms, early diagnosis, and interventional therapies (24). Any potential miRNA biomarkers must be capable of accurately distinguishing individuals with T1D from healthy controls. This specificity and sensitivity make miRNAs ideal candidate biomarkers, which could enhance the accuracy of diagnosis.

### 5.1 The application prospects of miRNAs in the diagnosis of T1D

Recent research findings indicate that cells can release free miRNAs under various physiological and pathological conditions. These extracellular miRNAs play a crucial role in intercellular communication, participating in the regulation of multiple biological processes, including angiogenesis, tumor cell invasion, and immune responses. Notably, these miRNA molecules exhibit significant resistance to degrading enzymes in the circulation and can accumulate persistently in specific tissues. This characteristic provides a solid theoretical basis for their potential application as disease biomarkers. Further studies are expected to reveal the important role of miRNAs in early disease diagnosis and prognosis assessment, thereby paving new avenues for the development of clinical medicine (25).

The goal of using miRNAs as biomarkers for T1D is to analyze their expression patterns to enable early disease prediction and identify the triggers of autoimmunity before autoantibody conversion. The progression of T1D is divided into four stages in clinical diagnosis (26). Stage 1 is characterized by the presence of multiple islet autoantibodies, normal blood glucose levels, and pre-symptomatic. Stage 2 involves multiple islet antibodies, raised blood glucose, pre-symptomatic. Stage 3 is marked by the Islet autoimmunity, raised blood glucose, symptomatic. Stage 4 represents long-standing T1D (27–30). There is a correlation between miRNAs and different stages of T1D. **Table 1** summarizes the miRNA expression profiles in individuals with recent-onset and long-standing T1D (31–40). MiR-25 is commonly found in both recent-onset and long-standing T1D individuals. In non-diabetic individuals with autoantibody positivity, miR-29, miR-21-3p, miR-491-5p, miR-150-5p, miR-204-5p, miR-339-3p, miR-148, and miR-425 are upregulated, while miR-497-5p is downregulated (35, 41). In recent-onset T1D patients, miR-152, miR-181a, and miR-27b are upregulated, while miR-375 is downregulated (42, 43). In long-standing T1D patients, miR-148a, miR-101-3p, miR-135a-5p, miR-143-3p, miR-223-3p, and miR-410-3p are upregulated, while miR-146a-5p and miR-495-3p are downregulated (39). These findings indicate that miRNA expression levels are closely related to autoimmune status and disease severity, highlighting their promising application in T1D diagnosis. Studies have shown that miR-200a-3p and miR-16-5p have high discriminatory power in the early diagnosis of T1D, effectively distinguishing between different stages of the disease. The expression levels of these two miRNAs are closely associated with disease progression, suggesting their potential as biomarkers for early identification of T1D (36).

**Table 1:** The miRNA expression profiles of patients with recent-onset and long-standing T1D.

Patient classification	miRNAs	Regulated	Function and role	Target Pathways/Genes	References
Recent-onset T1D	miR-152, miR-30a-5p, miR-181a, miR-24, miR-148a, miR-27a, miR-25, miR-200a, miR-122, miR-125b-5p, miR-136, miR-34a-5p, miR -342 -3p, miR-423-5p, miR-199a-3p, miR-126, miR -28 -5p, miR-454-3p, miR-222-3p, miR-144-5p	Up	Cell apoptosis, glucose control, islet homeostasis, and β-cell mass regulation.	The PTEN, aryl hydrocarbon receptor, STAT3, epithelial-mesenchymal transition, and senescence pathways.	(31–36)
Recent-onset T1D	miR-107, miR-16, miR-375	Down	Regulates insulin secretion and β cell mass.	PI3K/AKT.	(32, 35, 36)
Long-standing T1D (2–5 years duration)	miR-200a-3p, miR-346, miR-323a-3p, miR-874-3p, miR‐20b‐5p, miR‐190a‐5p, miR‐144‐5p, miR‐454‐3p, let‐7d‐5p, miR‐451a, miR‐425‐5p, miR‐103a‐3p, miR‐363‐3p, miR‐107	Up	miR-200a-3p is involved in NAD generation, antioxidant mechanisms, and nucleotide synthesis, which are associated with cell damage and repair.	TXNIP/miR-200/Zeb1/E-cadherin signaling pathway. Beta cell chaperone Dnajc3, caspase inhibitor XIAP, tumor suppressor Trp53.	(36, 37)
Long-standing T1D (2–5 years duration)	miR-16, miR-191, miR-125a-5p, miR-126, miR-374, miR-518d, miR-342-3p, miR-454, miR-146a, miR-155, miR-197, miR-483-5p	Down	miR-16 facilitates Treg induction and prevents apoptosis in β cells.	miR-16 targets CXCL10.	(36, 37)
Long-standing T1D (11.1 years duration)	miR-101-3p, miR-135a-5p, miR-143-3p, miR-223-3p, miR-410-3p, miR-21-5p, miR-148a	Up	Biomarkers for bone fragility, metabolism, resorption, and remodeling.	FoxO and TGF-β signaling pathways. VEGFA, IGF-1 receptor, AKT1.	(38, 39)
Long-standing T1D (11.1 years duration)	miR-495-3p	Down	Impact tissue development, inflammation, immunity, microalbuminuria, and glucose issues.	AKT1, VEGFA, and IGF-1 receptors.	(39)
Long-standing T1D (25 years duration)	miR-25-3p	Up	Glycemic control.	PTEN and Bcl-2.	(40)
Long-standing T1D (25 years duration)	miR-16, miR-302d-3p, miR-378e, miR-570-3p, miR-574-5p, miR-579	Down	miR-302 promotes insulin resistance in the liver, while miR-378 affects lipid metabolism and beta-cell death.	PI3K/AKT and miR-378 targets the IGF-1R.	(40)

Studies have revealed that certain miRNAs contribute to the progression of diabetes and the potential complications related to cardiovascular diseases in diabetic patients. For example, members of the miR-29 family are coordinately regulated in multiple tissues, including the heart and pancreas, and serve as early markers of diabetes. Under conditions of hyperinsulinemia, miR-29 family members are downregulated, but they increase sharply with the loss of hyperinsulinemia and elevated plasma glucose levels. To date, numerous studies have shown that exosomal miRNAs, such as miR-125b, miR-144, miR-155, miR-29, miR-133a, and miR-7, promote the onset and progression of diabetes. Additionally, miR-21 has been found to target the translation of the Bcl-2 gene, exacerbating β-cell apoptosis during diabetes progression (38, 44). miRNAs are also associated with the initiation of autoimmunity, β-cell dysfunction, and apoptosis, driving the development of T1D. For instance, miR-326 is highly expressed in peripheral blood lymphocytes of T1D patients, and its levels are highly correlated with persistent pancreatic autoimmunity and disease severity (45). miR-98, miR-23b, and miR-590-5p are overexpressed in CD8+ T cells derived from T1D patients, and the suppression of pro-apoptotic pathways by these miRNAs facilitates the unrestricted expansion of diabetogenic cytotoxic T cells (46). miR-510 is significantly upregulated in Tregs of T1D patients, while miR-342 and miR-191 are downregulated (47). miR-142-3p is induced during pancreatic autoimmunity, and the miR-142-3p/Tet2/Foxp3 axis impairs the differentiation and stability of Tregs in T1D models (48). Roggli et al. (49) reported that elevated expression of miR-21, miR-34, and miR-146 affects β-cell apoptosis.

### 5.2 miRNA-based interventions in T1D

In the treatment of T1D, the core objective is to halt the ongoing autoimmune response to protect the function of pancreatic β-cells, thereby maintaining normal glucose regulation. miRNAs, as a class of critical molecules, play an important role in the pathogenesis and progression of T1D (50). They modulate disease progression by regulating the expression levels of target proteins. In fact, alterations in miRNA expression are closely associated with the development of T1D, providing a theoretical basis for miRNA-based interventions (51). On one hand, restoring the normal expression of specific miRNAs by using miRNA mimics or inhibiting their activity may emerge as a novel therapeutic strategy for T1D. On the other hand, inducing the differentiation of pluripotent stem cells into pancreatic β-cells represents a potential therapeutic avenue. In this process, miRNAs such as miR-375, miR-7, miR-21, and miR-29 play crucial roles in regulating signaling pathways, offering new hope for T1D treatment (52–54). With the development of personalized medicine and the application of advanced diagnostic and prognostic technologies, healthcare professionals can gain a deeper understanding of the complex mechanisms underlying T1D. This enables them to provide more precise and personalized clinical assessments and treatment plans for each patient, which is of great significance for improving the prognosis of individuals with T1D.

#### 5.2.1 miRNAs and diabetic nephropathy

miRNAs are closely associated with the development of diabetic nephropathy (DN) (55). Studies have identified that miR-21, miR-377, miR-93, and miR-216a may be involved in the pathogenesis of DN, while miR-25 appears to have a protective effect on the kidneys (56, 57). The regulation of cellular activity relies on the core mechanisms of phosphorylation and dephosphorylation, which play important roles in stimulating cell growth. Phosphatase and tensin homolog (PTEN), located on chromosome 10, catalyzes the dephosphorylation of phosphatidylinositol 3,4,5-trisphosphate (PI3,4,5-P3). This leads to the inactivation of Akt kinase. Thus, the concentration of PTEN is closely linked to the degree of Akt inactivation, and it further affects the accumulation of extracellular matrix proteins through the regulation of downstream signaling pathways (58). miR-21 is one of the most important miRNAs involved in renal fibrosis, acting on inflammation, angiogenesis, and immune destruction (59, 60). Research has shown that in the renal cortex of OVE26 T1D mice, the expression level of miR-21 is significantly increased, which is closely related to the decreased levels of PTEN and the increased secretion of fibronectin (24). Specifically, high glucose levels induce elevated miR-21 secretion in renal cells, which suppresses the expression of PRAS40, enhances TORC1 activity, and leads to renal cell hypertrophy and increased fibronectin expression. miR-21 can also stimulate glomerular mesangial cells to secrete miR-21, which targets the 3’-UTR of PTEN mRNA, inhibits PTEN expression, and results in increased Akt phosphorylation levels. Studies have also found that the upregulation of miR-377 in DN patients is associated with the pathogenesis of diabetic microvascular and macrovascular complications. Therefore, inhibiting miR-377 expression has been explored as a novel therapeutic approach for DN (61–63). Additionally, miR-25 is downregulated in DN patients and negatively correlates with the albumin-to-creatinine ratio (ACR), suggesting that it may exert protective effects on the kidneys by activating the PTEN/Akt pathway, acting as an antioxidant and anti-apoptotic agent (64–66). In early-stage DN patients, serum miR-29c is found to be downregulated, and its low expression can exacerbate the fibrotic process, indicating its potential value for early intervention in renal disease (67). miRNA-based therapies aim to reverse early pathological changes by inhibiting harmful miRNAs, TGF-β, and fibronectin accumulation. This reduces renal fibrosis and inflammation, thereby preventing the progression to end-stage renal disease and improving the quality of life for DN patients.

#### 5.2.2 miRNAs and diabetic retinopathy

Diabetic retinopathy (DR) is a common complication in patients with T1D. It is also one of the leading causes of irreversible blindness in the working-age population worldwide (68, 69). In T1D, the downregulation of miR-126 is negatively correlated with the occurrence of multiple complications, especially proliferative diabetic retinopathy (PDR). Studies have shown that miR-126 regulates the expression of vascular endothelial growth factor (VEGF). This regulation influences angiogenesis and the progression of retinopathy. Additionally, miR-200b may exert protective effects in DR by regulating the expression of the antioxidant gene Oxr1. These findings suggest that miR-126 and miR-200b play important roles in the intervention and control of DR and may serve as potential therapeutic targets. Oza et al. (70) found that hypertension and advanced age are significant risk factors for DR. Therefore, blood pressure management in children and adolescents with T1D may help reduce the incidence of DR. Moreover, improved glycemic control can effectively slow the progression of DR.

#### 5.2.3 miRNAs and diabetic cardiomyopathy

Studies have revealed that in a streptozotocin-induced diabetic mouse model, the expression of miR-144 is downregulated. Inhibition of miR-144 effectively blocks the generation of reactive oxygen species (ROS) and cardiomyocyte apoptosis triggered by high glucose levels. Although miR-144 is not the sole factor contributing to increased oxidative stress, its inhibition has shown potential clinical value in alleviating cardiac oxidative stress and protecting myocardial function (71). Knockdown of miR-195 has been shown to improve coronary blood flow and myocardial function, reduce myocardial hypertrophy, and highlight its potential as a novel therapeutic target for diabetic cardiomyopathy through mechanisms such as promoting angiogenesis, inhibiting apoptosis, and reducing oxidative damage (72). Further research has identified that in the hearts of diabetic mice treated with low-dose streptozotocin, 29 miRNAs are differentially expressed, with miR-141 being particularly significant. miR-141 directly regulates solute carrier family 25 member 3 (Slc25a3), which affects mitochondrial ATP synthesis. This indicates that diabetes-induced changes in miRNA expression have profound impacts on mitochondrial function and ATP synthesis, underscoring the important role of miRNAs in the treatment of diabetic cardiomyopathy (73).

## 6 Discussion

Currently, the treatment of T1D and its complications imposes a heavy economic burden on both society and individuals (74). Early identification of high-risk populations and the implementation of preventive measures are crucial for avoiding or delaying disease progression. Given the unclear etiology of T1D, there is an urgent need to explore new biomarkers and their roles in disease development. From a clinical perspective, these emerging biomarkers are expected to facilitate early detection of T1D and promote more precise treatment strategies, thereby significantly improving patients’ quality of life. miRNAs have shown great potential as biomarkers. They are stable in body fluids, resistant to ribonucleases and repeated freeze-thaw cycles, and detectable through highly sensitive and specific quantitative techniques (75, 76). In the clinical management of T1D, miRNAs have demonstrated significant advantages by targeting specific pathological processes. Moreover, their multitarget nature allows them to affect multiple pathological pathways simultaneously, enhancing therapeutic efficacy. As key regulators of β-cells, miRNAs are involved in multiple mechanisms, including the immune system, β-cell differentiation, function, survival, and responses to viral infections. Their dysregulation can drive the progression of T1D. As key regulators of β-cells, miRNAs are involved in multiple mechanisms. These include the immune system, β-cell differentiation, function, survival, and responses to viral infections. A comprehensive exploration of miRNAs will shed light on the pathogenesis of T1D and pave the way for the development of innovative therapeutic strategies. For example, upregulation of miR-135a may directly or indirectly influence the development of renal fibrosis in diabetic patients, making miR-135a a potential therapeutic target for diabetic nephropathy in clinical research (77). However, miRNAs such as miR-409-3p are expressed at low levels in individuals with T1D. They have the potential to monitor therapeutic interventions but have not yet been studied in at-risk populations. Insulitis remains a key pathological factor in T1D but is not the sole cause, which is closely related to the design of clinical trials targeting pathogenic mechanisms more effectively. Combination therapies aimed at promoting immune regulation and addressing β-cell dysfunction may be more effective in treating this chronic disease. miRNAs can serve as early biomarkers in the clinical context of T1D, providing a personalized approach to reducing long-term complications associated with T1D.

## 7 Challenges and future outlook

In the early diagnosis and intervention of T1D, the application of miRNAs faces numerous challenges. First, the regulatory mechanisms of miRNAs are highly complex, with each miRNA potentially targeting multiple genes and multiple miRNAs regulating the same gene. This intricate network makes it difficult to elucidate the precise roles of miRNAs (78). MiRNAs act primarily as gene expression regulators in the pathogenesis of T1D by inhibiting translation or inducing mRNA degradation, thereby hindering protein synthesis at the post-transcriptional level. Additionally, miRNAs may trigger intense oxidative stress that exceeds the capacity of cellular antioxidant responses, leading to the accumulation of reactive oxygen species (ROS). This process can result in pancreatic β-cell dysfunction and impaired glucose tolerance, both of which significantly impact the pathogenesis of T1D (25, 79). Additionally, the interactions between miRNAs and other epigenetic factors further complicate miRNA regulation. Third, miRNA detection requires stringent sample handling and timely analysis, ideally within 8 hours of sample collection. However, timely transport and processing of samples are often challenging in most clinical settings, limiting the widespread use of miRNAs as biomarkers (80). Fourth, miRNA-based therapies may trigger off-target effects, leading to nonspecific actions and potential toxicity, which can impact treatment safety (81). Finally, the currently identified miRNA biomarkers lack sufficient specificity to fully replace existing diagnostic methods. These challenges indicate that although miRNAs hold potential value in the early diagnosis and intervention of T1D, significant breakthroughs are still needed in target validation and clinical trial design to realize their clinical application. Future research will further elucidate the mechanisms and application value of miRNAs in T1D, with the potential to drive their widespread use in early diagnosis and intervention.

## References

[1] Antar SA Ashour NA Sharaky M Khattab M Ashour NA Zaid RT. Diabetes mellitus: Classification, mediators, and complications; A gate to identify potential targets for the development of new effective treatments. BioMed Pharmacother. (2023) 168:115734. doi: 10.1016/j.biopha.2023.115734 37857245

[2] ElSayed NA Aleppo G Aroda VR Bannuru RR Brown FM Bruemmer D. 2. Classification and diagnosis of diabetes: standards of care in diabetes-2023. Diabetes Care. (2023) 46:S19–s40. doi: 10.2337/dc23-S002 36507649 PMC9810477

[3] Mayer-Davis EJ Lawrence JM Dabelea D Divers J Isom S Dolan L. Incidence trends of type 1 and type 2 diabetes among youths, 2002-2012. N Engl J Med. (2017) 376:1419–29. doi: 10.1056/NEJMoa1610187 PMC5592722 28402773

[4] Gregory GA Robinson TIG Linklater SE Wang F Colagiuri S de Beaufort C. Global incidence, prevalence, and mortality of type 1 diabetes in 2021 with projection to 2040: a modelling study. Lancet Diabetes Endocrinol. (2022) 10:741–60. doi: 10.1016/s2213-8587(22)00218-2 36113507

[5] Taheri M Eghtedarian R Dinger ME Ghafouri-Fard S. Emerging roles of non-coding RNAs in the pathogenesis of type 1 diabetes mellitus. BioMed Pharmacother. (2020) 129:110509. doi: 10.1016/j.biopha.2020.110509 32768981

[6] Auddino S Aiello E Grieco GE Dotta F Sebastiani G. A three-layer perspective on miRNA regulation in β cell inflammation. Trends Endocrinol Metab. (2024) 24:15. doi: 10.1016/j.tem.2024.10.002 39532586

[7] Saxby N Beggs S Kariyawasam N Battersby M Lawn S. Do guidelines provide evidence-based guidance to health professionals on promoting developmentally appropriate chronic condition self-management in children? A systematic review. Chronic Illn. (2020) 16:239–52. doi: 10.1177/1742395318799844 30244592

[8] Lee JJ Thompson MJ Usher-Smith JA Koshiaris C Van den Bruel A. Opportunities for earlier diagnosis of type 1 diabetes in children: A case-control study using routinely collected primary care records. Prim Care Diabetes. (2018) 12:254–64. doi: 10.1016/j.pcd.2018.02.002 29548694

[9] Fazeli Farsani S Brodovicz K Soleymanlou N Marquard J Wissinger E Maiese BA. Incidence and prevalence of diabetic ketoacidosis (DKA) among adults with type 1 diabetes mellitus (T1D): a systematic literature review. BMJ Open. (2017) 7:e016587. doi: 10.1136/bmjopen-2017-016587 PMC5642652 28765134

[10] Powers AC. Type 1 diabetes mellitus: much progress, many opportunities. J Clin Invest. (2021) 131:e142242. doi: 10.1172/jci142242 33759815 PMC8262558

[11] Singh R Gholipourmalekabadi M Shafikhani SH. Animal models for type 1 and type 2 diabetes: advantages and limitations. Front Endocrinol (Lausanne). (2024) 15:1359685. doi: 10.3389/fendo.2024.1359685 38444587 PMC10912558

[12] Xie Z Chang C Huang G Zhou Z. The role of epigenetics in type 1 diabetes. Adv Exp Med Biol. (2020) 1253:223–57. doi: 10.1007/978-981-15-3449-2_9 32445098

[13] Basina M Maahs DM. Age at type 1 diabetes onset: a new risk factor and call for focused treatment. Lancet. (2018) 392:453–4. doi: 10.1016/s0140-6736(18)31811-7 30129445

[14] Basu M Pandit K Banerjee M Mondal SA Mukhopadhyay P Ghosh S. Profile of auto-antibodies (Disease related and other) in children with type 1 diabetes. Indian J Endocrinol Metab. (2020) 24:256–9. doi: 10.4103/ijem.IJEM_63_20 PMC7539037 33083265

[15] Allen DW Kim KW Rawlinson WD Craig ME. Maternal virus infections in pregnancy and type 1 diabetes in their offspring: Systematic review and meta-analysis of observational studies. Rev Med Virol. (2018) 28:e1974. doi: 10.1002/rmv.1974 29569297

[16] Chapman NM Coppieters K von Herrath M Tracy S. The microbiology of human hygiene and its impact on type 1 diabetes. Islets. (2012) 4:253–61. doi: 10.4161/isl.21570 PMC3496651 22996796

[17] Hamilton-Williams EE Lorca GL Norris JM Dunne JL. A triple threat? The role of diet, nutrition, and the microbiota in T1D pathogenesis. Front Nutr. (2021) 8:600756. doi: 10.3389/fnut.2021.600756 33869260 PMC8046917

[18] Sims EK DiMeglio LA. Cause or effect? A review of clinical data demonstrating beta cell dysfunction prior to the clinical onset of type 1 diabetes. Mol Metab. (2019) 27s:S129–s38. doi: 10.1016/j.molmet.2019.06.010 PMC6768572 31500824

[19] Austin MC Muralidharan C Roy S Crowder JJ Piganelli JD Linnemann AK. Dysfunctional β-cell autophagy induces β-cell stress and enhances islet immunogenicity. Front Immunol. (2025) 16:1504583. doi: 10.3389/fimmu.2025.1504583 39944686 PMC11814175

[20] Scherm MG Daniel C. miRNA-mediated immune regulation in islet autoimmunity and type 1 diabetes. Front Endocrinol (Lausanne). (2020) 11:606322. doi: 10.3389/fendo.2020.606322 33329406 PMC7731293

[21] Ang L Gunaratnam S Huang Y Dillon BR Martin CL Burant A. Inflammatory markers and measures of cardiovascular autonomic neuropathy in type 1 diabetes. J Am Heart Assoc. (2025) 14:e036787. doi: 10.1161/jaha.124.036787 39727210 PMC12054404

[22] Williams CL Aitken RJ Wilson IV Mortimer GLM Long AE Williams AJK. The measurement of autoantibodies to insulin informs diagnosis of diabetes in a childhood population negative for other autoantibodies. Diabetes Med. (2022) 39:e14979. doi: 10.1111/dme.14979 PMC9827938 36251483

[23] Yi L Swensen AC Qian WJ. Serum biomarkers for diagnosis and prediction of type 1 diabetes. Transl Res. (2018) 201:13–25. doi: 10.1016/j.trsl.2018.07.009 30144424 PMC6177288

[24] Miao C Chang J Zhang G Fang Y. MicroRNAs in type 1 diabetes: new research progress and potential directions. Biochem Cell Biol. (2018) 96:498–506. doi: 10.1139/bcb-2018-0027 29554441

[25] Fyvie MJ Gillespie KM. The importance of biomarker development for monitoring type 1 diabetes progression rate and therapeutic responsiveness. Front Immunol. (2023) 14:1158278. doi: 10.3389/fimmu.2023.1158278 37256143 PMC10225507

[26] Insel RA Dunne JL Atkinson MA Chiang JL Dabelea D Gottlieb PA. Staging presymptomatic type 1 diabetes: a scientific statement of JDRF, the Endocrine Society, and the American Diabetes Association. Diabetes Care. (2015) 38:1964–74. doi: 10.2337/dc15-1419 PMC5321245 26404926

[27] Roberts MS Burbelo PD Egli-Spichtig D Perwad F Romero CJ Ichikawa S. Autoimmune hyperphosphatemic tumoral calcinosis in a patient with FGF23 autoantibodies. J Clin Invest. (2018) 128:5368–73. doi: 10.1172/jci122004 PMC6264742 30226830

[28] Pozzilli P Pieralice S. Latent autoimmune diabetes in adults: current status and new horizons. Endocrinol Metab (Seoul). (2018) 33:147–59. doi: 10.3803/EnM.2018.33.2.147 PMC6021307 29947172

[29] Couper JJ Haller MJ Greenbaum CJ Ziegler AG Wherrett DK Knip M. ISPAD Clinical Practice Consensus Guidelines 2018: Stages of type 1 diabetes in children and adolescents. Pediatr Diabetes. (2018) 19 Suppl 27:20–7. doi: 10.1111/pedi.12734 30051639

[30] Sims EK Besser REJ Dayan C Geno Rasmussen C Greenbaum C Griffin KJ. Screening for type 1 diabetes in the general population: A status report and perspective. Diabetes. (2022) 71:610–23. doi: 10.2337/dbi20-0054 PMC9114719 35316839

[31] Nielsen LB Wang C Sørensen K Bang-Berthelsen CH Hansen L Andersen ML. Circulating levels of microRNA from children with newly diagnosed type 1 diabetes and healthy controls: evidence that miR-25 associates to residual beta-cell function and glycaemic control during disease progression. Exp Diabetes Res. (2012) 2012:896362. doi: 10.1155/2012/896362 22829805 PMC3398606

[32] Marchand L Jalabert A Meugnier E Van den Hende K Fabien N Nicolino M. miRNA-375 a sensor of glucotoxicity is altered in the serum of children with newly diagnosed type 1 diabetes. J Diabetes Res. (2016) 2016:1869082. doi: 10.1155/2016/1869082 27314045 PMC4895032

[33] Erener S Marwaha A Tan R Panagiotopoulos C Kieffer TJ. Profiling of circulating microRNAs in children with recent onset of type 1 diabetes. JCI Insight. (2017) 2:e89656. doi: 10.1172/jci.insight.89656 28239651 PMC5313074

[34] Liu L Yan J Xu H Zhu Y Liang H Pan W. Two novel microRNA biomarkers related to β-cell damage and their potential values for early diagnosis of type 1 diabetes. J Clin Endocrinol Metab. (2018) 103:1320–9. doi: 10.1210/jc.2017-01417 29370422

[35] Åkerman L Casas R Ludvigsson J Tavira B Skoglund C. Serum miRNA levels are related to glucose homeostasis and islet autoantibodies in children with high risk for type 1 diabetes. PloS One. (2018) 13:e0191067. doi: 10.1371/journal.pone.0191067 29346396 PMC5773164

[36] Santos AS Ferreira LRP da Silva AC Alves LI Damasceno JG Kulikowski L. Progression of type 1 diabetes: circulating microRNA expression profiles changes from preclinical to overt disease. J Immunol Res. (2022) 2022:2734490. doi: 10.1155/2022/2734490 35903753 PMC9325579

[37] Pang H Fan W Pi L Shi X Wang Z Luo S. Plasma-derived exosomal miRNA profiles associated with type 1 diabetes. Diabetes Metab Res Rev. (2024) 40:e3774. doi: 10.1002/dmrr.3774 38340050

[38] Grieco GE Cataldo D Ceccarelli E Nigi L Catalano G Brusco N. Serum levels of miR-148a and miR-21-5p are increased in type 1 diabetic patients and correlated with markers of bone strength and metabolism. Noncoding RNA. (2018) 4:37–53. doi: 10.3390/ncrna4040037 PMC6315714 30486455

[39] Swolin-Eide D Forsander G Pundziute Lyckå A Novak D Grillari J Diendorfer AB. Circulating microRNAs in young individuals with long-duration type 1 diabetes in comparison with healthy controls. Sci Rep. (2023) 13:11634. doi: 10.1038/s41598-023-38615-7 37468555 PMC10356803

[40] Garcia-Contreras M Shah SH Tamayo A Robbins PD Golberg RB Mendez AJ. Plasma-derived exosome characterization reveals a distinct microRNA signature in long duration Type 1 diabetes. Sci Rep. (2017) 7:5998. doi: 10.1038/s41598-017-05787-y 28729721 PMC5519761

[41] Snowhite IV Allende G Sosenko J Pastori RL Messinger Cayetano S Pugliese A. Association of serum microRNAs with islet autoimmunity, disease progression and metabolic impairment in relatives at risk of type 1 diabetes. Diabetologia. (2017) 60:1409–22. doi: 10.1007/s00125-017-4294-3 PMC5839115 28500393

[42] Samandari N Mirza AH Nielsen LB Kaur S Hougaard P Fredheim S. Circulating microRNA levels predict residual beta cell function and glycaemic control in children with type 1 diabetes mellitus. Diabetologia. (2017) 60:354–63. doi: 10.1007/s00125-016-4156-4 27866223

[43] Liu Y Ma M Yu J Ping F Zhang H Li W. Decreased Serum microRNA-21, microRNA-25, microRNA-146a, and microRNA-181a in Autoimmune Diabetes: Potential Biomarkers for Diagnosis and Possible Involvement in Pathogenesis. Int J Endocrinol. (2019) 2019:8406438. doi: 10.1155/2019/8406438 31582977 PMC6754900

[44] Lakhter AJ Pratt RE Moore RE Doucette KK Maier BF DiMeglio LA. Beta cell extracellular vesicle miR-21-5p cargo is increased in response to inflammatory cytokines and serves as a biomarker of type 1 diabetes. Diabetologia. (2018) 61:1124–34. doi: 10.1007/s00125-018-4559-5 PMC5878132 29445851

[45] Sebastiani G Grieco FA Spagnuolo I Galleri L Cataldo D Dotta F. Increased expression of microRNA miR-326 in type 1 diabetic patients with ongoing islet autoimmunity. Diabetes Metab Res Rev. (2011) 27:862–6. doi: 10.1002/dmrr.1262 22069274

[46] de Jong VM van der Slik AR Laban S van ‘t Slot R Koeleman BP Zaldumbide A. Survival of autoreactive T lymphocytes by microRNA-mediated regulation of apoptosis through TRAIL and Fas in type 1 diabetes. Genes Immun. (2016) 17:342–8. doi: 10.1038/gene.2016.29 27467285

[47] Hezova R Slaby O Faltejskova P Mikulkova Z Buresova I Raja KR. microRNA-342, microRNA-191 and microRNA-510 are differentially expressed in T regulatory cells of type 1 diabetic patients. Cell Immunol. (2010) 260:70–4. doi: 10.1016/j.cellimm.2009.10.012 19954774

[48] Scherm MG Serr I Zahm AM Schug J Bellusci S Manfredini R. miRNA142-3p targets Tet2 and impairs Treg differentiation and stability in models of type 1 diabetes. Nat Commun. (2019) 10:5697. doi: 10.1038/s41467-019-13587-3 31836704 PMC6910913

[49] Margaritis K Margioula-Siarkou G Giza S Kotanidou EP Tsinopoulou VR Christoforidis A. Micro-RNA implications in type-1 diabetes mellitus: A review of literature. Int J Mol Sci. (2021) 22:12165. doi: 10.3390/ijms222212165 34830046 PMC8621893

[50] Wang Z Xie Z Lu Q Chang C Zhou Z. Beyond genetics: what causes type 1 diabetes. Clin Rev Allergy Immunol. (2017) 52:273–86. doi: 10.1007/s12016-016-8592-1 27878451

[51] Ventriglia G Mancarella F Sebastiani G Cook DP Mallone R Mathieu C. miR-409-3p is reduced in plasma and islet immune infiltrates of NOD diabetic mice and is differentially expressed in people with type 1 diabetes. Diabetologia. (2020) 63:124–36. doi: 10.1007/s00125-019-05026-1 31659408

[52] Lahmy R Soleimani M Sanati MH Behmanesh M Kouhkan F Mobarra N. MiRNA-375 promotes beta pancreatic differentiation in human induced pluripotent stem (hiPS) cells. Mol Biol Rep. (2014) 41:2055–66. doi: 10.1007/s11033-014-3054-4 24469711

[53] Shaer A Azarpira N Karimi MH Soleimani M Dehghan S. Differentiation of human-induced pluripotent stem cells into insulin-producing clusters by microRNA-7. Exp Clin Transplant. (2016) 14:555–63. doi: 10.6002/ect.2014.0144 26103160

[54] Pileggi A Klein D Fotino C Bravo-Egaña V Rosero S Doni M. MicroRNAs in islet immunobiology and transplantation. Immunol Res. (2013) 57:185–96. doi: 10.1007/s12026-013-8436-5 24242759

[55] Zhong X Chung AC Chen HY Dong Y Meng XM Li R. miR-21 is a key therapeutic target for renal injury in a mouse model of type 2 diabetes. Diabetologia. (2013) 56:663–74. doi: 10.1007/s00125-012-2804-x 23292313

[56] Erener S Ellis CE Ramzy A Glavas MM O’Dwyer S Pereira S. Deletion of pancreas-specific miR-216a reduces beta-cell mass and inhibits pancreatic cancer progression in mice. Cell Rep Med. (2021) 2:100434. doi: 10.1016/j.xcrm.2021.100434 34841287 PMC8606901

[57] Wang P Liu Q Zhao H Bishop JO Zhou G Olson LK. miR-216a-targeting theranostic nanoparticles promote proliferation of insulin-secreting cells in type 1 diabetes animal model. Sci Rep. (2020) 10:5302. doi: 10.1038/s41598-020-62269-4 32210316 PMC7093482

[58] Ngeow J Sesock K Eng C. Clinical implications for germline PTEN spectrum disorders. Endocrinol Metab Clin North Am. (2017) 46:503–17. doi: 10.1016/j.ecl.2017.01.013 28476234

[59] Fouad M Salem I Elhefnawy K Raafat N Faisal A. MicroRNA-21 as an early marker of nephropathy in patients with type 1 diabetes. Indian J Nephrol. (2020) 30:21–5. doi: 10.4103/ijn.IJN_80_19 PMC6977383 32015595

[60] Kölling M Kaucsar T Schauerte C Hübner A Dettling A Park JK. Therapeutic miR-21 silencing ameliorates diabetic kidney disease in mice. Mol Ther. (2017) 25:165–80. doi: 10.1016/j.ymthe.2016.08.001 PMC5363308 28129112

[61] Abdelghaffar S Shora H Abdelatty S Elmougy F El Sayed R Abdelrahman H. MicroRNAs and risk factors for diabetic nephropathy in Egyptian children and adolescents with type 1 diabetes. Diabetes Metab Syndr Obes. (2020) 13:2485–94. doi: 10.2147/dmso.S247062 PMC7367734 32765027

[62] Wu R Niu Z Ren G Ruan L Sun L. CircSMAD4 alleviates high glucose-induced inflammation, extracellular matrix deposition and apoptosis in mouse glomerulus mesangial cells by relieving miR-377-3p-mediated BMP7 inhibition. Diabetol Metab Syndr. (2021) 13(1):137. doi: 10.1186/s13098-021-00753-1 34801077 PMC8606083

[63] El-Samahy MH Adly AA Elhenawy YI Ismail EA Pessar SA Mowafy ME. Urinary miRNA-377 and miRNA-216a as biomarkers of nephropathy and subclinical atherosclerotic risk in pediatric patients with type 1 diabetes. J Diabetes Complications. (2018) 32:185–92. doi: 10.1016/j.jdiacomp.2017.10.014 29175120

[64] Li H Zhu X Zhang J Shi J. MicroRNA-25 inhibits high glucose-induced apoptosis in renal tubular epithelial cells via PTEN/AKT pathway. BioMed Pharmacother. (2017) 96:471–9. doi: 10.1016/j.biopha.2017.10.019 29031207

[65] Gholaminejad A Abdul Tehrani H Gholami Fesharaki M. Identification of candidate microRNA biomarkers in diabetic nephropathy: a meta-analysis of profiling studies. J Nephrol. (2018) 31:813–31. doi: 10.1007/s40620-018-0511-5 30019103

[66] Liu Y Li H Liu J Han P Li X Bai H. Variations in microRNA-25 expression influence the severity of diabetic kidney disease. J Am Soc Nephrol. (2017) 28:3627–38. doi: 10.1681/asn.2015091017 PMC5698056 28923913

[67] Sun CM Zhang WY Wang SY Qian G Pei DL Zhang GM. Fer exacerbates renal fibrosis and can be targeted by miR-29c-3p. Open Med (Wars). (2021) 16:1378–85. doi: 10.1515/med-2021-0319 PMC8439263 34595351

[68] Montaser E Shah VN. Prediction of incident diabetic retinopathy in adults with type 1 diabetes using machine learning approach: an exploratory study. J Diabetes Sci Technol. (2024), 1–8. doi: 10.1177/19322968241292369 PMC11571610 39465559

[69] Paliares IC Dualib PM Torres LSN Aroucha PMT de Almeida-Pititto B de Sa JR. Evaluation of the Steno Type 1 Risk Engine in predicting cardiovascular events in an ethnic mixed population of type 1 diabetes mellitus and its association with chronic microangiopathy complications. Cardiovasc Diabetol. (2024) 23:374. doi: 10.1186/s12933-024-02460-3 39438880 PMC11515709

[70] Oza C Khadilkar A Bhor S Curran K Sambare C Ladkat D. Prevalence and predictors of diabetic retinopathy, its progression and regression in Indian children and youth with type-1 diabetes. Clin Med Insights Endocrinol Diabetes. (2024) 17:11795514241275921. doi: 10.1177/11795514241275921 39233877 PMC11372767

[71] Yu M Liu Y Zhang B Shi Y Cui L Zhao X. Inhibiting microRNA-144 abates oxidative stress and reduces apoptosis in hearts of streptozotocin-induced diabetic mice. Cardiovasc Pathol. (2015) 24:375–81. doi: 10.1016/j.carpath.2015.06.003 26164195

[72] Zheng D Ma J Yu Y Li M Ni R Wang G. Silencing of miR-195 reduces diabetic cardiomyopathy in C57BL/6 mice. Diabetologia. (2015) 58:1949–58. doi: 10.1007/s00125-015-3622-8 PMC4499474 25994075

[73] Baseler WA Thapa D Jagannathan R Dabkowski ER Croston TL Hollander JM. miR-141 as a regulator of the mitochondrial phosphate carrier (Slc25a3) in the type 1 diabetic heart. Am J Physiol Cell Physiol. (2012) 303:C1244–51. doi: 10.1152/ajpcell.00137.2012 PMC3532490 23034391

[74] Rodríguez-Muñoz A Picón-César MJ Tinahones FJ Martínez-Montoro JI. Type 1 diabetes-related distress: Current implications in care. Eur J Intern Med. (2024) 125:19–27. doi: 10.1016/j.ejim.2024.03.030 38609810

[75] Nam YH Kwak T Kim H Jeon S Bae S Yang Y. A new quantitative real-time PCR method to measure human miRNAs using the PROMER technology. Biochem Biophys Res Commun. (2024) 741:151069. doi: 10.1016/j.bbrc.2024.151069 39616937

[76] Li Y Chen S Rao H Cui S Chen G. MicroRNA gets a mighty award. Adv Sci (Weinh). (2025) 12:e2414625. doi: 10.1002/advs.202414625 39836690 PMC11831481

[77] Zhang J Zhang L Zha D Wu X. Inhibition of miRNA−135a−5p ameliorates TGF−β1−induced human renal fibrosis by targeting SIRT1 in diabetic nephropathy. Int J Mol Med. (2020) 46:1063–73. doi: 10.3892/ijmm.2020.4647 PMC7387088 32705273

[78] Mustafa R Mens MMJ van Hilten A Huang J Roshchupkin G Huan T. A comprehensive study of genetic regulation and disease associations of plasma circulatory microRNAs using population-level data. Genome Biol. (2024) 25:276. doi: 10.1186/s13059-024-03420-6 39434104 PMC11492503

[79] Qadir MMF Klein D Álvarez-Cubela S Domínguez-Bendala J Pastori RL. The role of microRNAs in diabetes-related oxidative stress. Int J Mol Sci. (2019) 20:5423. doi: 10.3390/ijms20215423 31683538 PMC6862492

[80] Cacheux J Bancaud A Leichlé T Cordelier P. Technological challenges and future issues for the detection of circulating microRNAs in patients with cancer. Front Chem. (2019) 7:815. doi: 10.3389/fchem.2019.00815 31850308 PMC6894013

[81] Abdelaal AM Sohal IS Iyer S Sudarshan K Kothandaraman H Lanman NA. A first-in-class fully modified version of miR-34a with outstanding stability, activity, and anti-tumor efficacy. Oncogene. (2023) 42:2985–99. doi: 10.1038/s41388-023-02801-8 PMC10541324 37666938

